# Tissue Engineering of Urinary Bladder and Urethra: Advances from Bench to Patients

**DOI:** 10.1155/2013/154564

**Published:** 2013-12-24

**Authors:** Hazem Orabi, Sara Bouhout, Amélie Morissette, Alexandre Rousseau, Stéphane Chabaud, Stéphane Bolduc

**Affiliations:** ^1^Centre LOEX de l'Université Laval, Génie Tissulaire et Régénératrice, LOEX du Centre de Recherche FRQS du Centre de Recherche de CHU de Québec, Axe Médecine Régénératrice, Aile-R Centre Hospitalier Affilié Universitaire de Québec, 1401 18e rue, Québec, QC, Canada G1J 1Z4; ^2^Département de Chirurgie, Faculté de Médecine, Université Laval, Québec, QC, Canada G1K 7P4

## Abstract

Urinary tract is subjected to many varieties of pathologies since birth including congenital anomalies, trauma, inflammatory lesions, and malignancy. These diseases necessitate the replacement of involved organs and tissues. Shortage of organ donation, problems of immunosuppression, and complications associated with the use of nonnative tissues have urged clinicians and scientists to investigate new therapies, namely, tissue engineering. Tissue engineering follows principles of cell transplantation, materials science, and engineering. Epithelial and muscle cells can be harvested and used for reconstruction of the engineered grafts. These cells must be delivered in a well-organized and differentiated condition because water-seal epithelium and well-oriented muscle layer are needed for proper function of the substitute tissues. Synthetic or natural scaffolds have been used for engineering lower urinary tract. Harnessing autologous cells to produce their own matrix and form scaffolds is a new strategy for engineering bladder and urethra. This self-assembly technique avoids the biosafety and immunological reactions related to the use of biodegradable scaffolds. Autologous equivalents have already been produced for pigs (bladder) and human (urethra and bladder). The purpose of this paper is to present a review for the existing methods of engineering bladder and urethra and to point toward perspectives for their replacement.

## 1. Introduction

Lower urinary tract is composed of urinary bladder (UB), urethra, and urinary sphincters. It is responsible for urine storage and its evacuation. In addition, in men, the urethra is also used by the seminal ducts and carries the sperm from the verumontanum to the external urethral orifice [1]. The review will be concerned with tissue engineering of bladder and urethra only.

Many pathologies affect the urinary bladder and urethra and hence health and quality of life of the patients at different ages and sexes and demand their replacement. These diseases have high incidence and long-term impact, which increase the burden of health systems all over the world. The main necessities for bladder surgical reconstruction are vesical exstrophy, neurogenic bladders, contracted bladder, and urothelial carcinoma. The gold standard technique for bladder replacement is the use of intestinal segments [2]. Since the intestine is structurally and functionally different from urinary bladder, many complications exist [3, 4] such as hypocontractility, hematuria, dysuria, urolithiasis, neoplasia, ectopic mucus production, and metabolic imbalances due to urine absorption by the intestinal mucosa. The latter can induce delay of growth and reduction of bone density in pediatric patients [5–8].

Various urethral conditions, such as inflammatory and posttraumatic strictures, congenital defects, and malignancy, often require extensive urethral reconstruction. Currently, they are treated with autologous graft or flap from genital skin or buccal mucosa [9]. There may be a limited donor supply of tissues needed for long segment replacement. No matter how good the initial result is, on the long term—more than 10 years—all skin tubes (from genital or extragenital sources, whether used as grafts or flaps) seem to have a tendency to deteriorate [10]. Additionally, there are problems of tissue impairment and morbidity caused by harvesting buccal mucosa and lack of long graft [11]. When used in a staged procedure, the buccal mucosa graft does not heal in the same way in all patients, and numerous revisions of the graft bed could be necessary to obtain a satisfactory mucosal bed before urethral closure [12].

That is why the field of tissue engineering and regenerative medicine has evolved to compensate for the replacement of these organs to prevent complications and improve the quality of life for patients suffering from major diseases necessitating bladder and urethral substitution.

## 2. Anatomical Considerations of Urinary Bladder and Urethra

**Figure 1:**
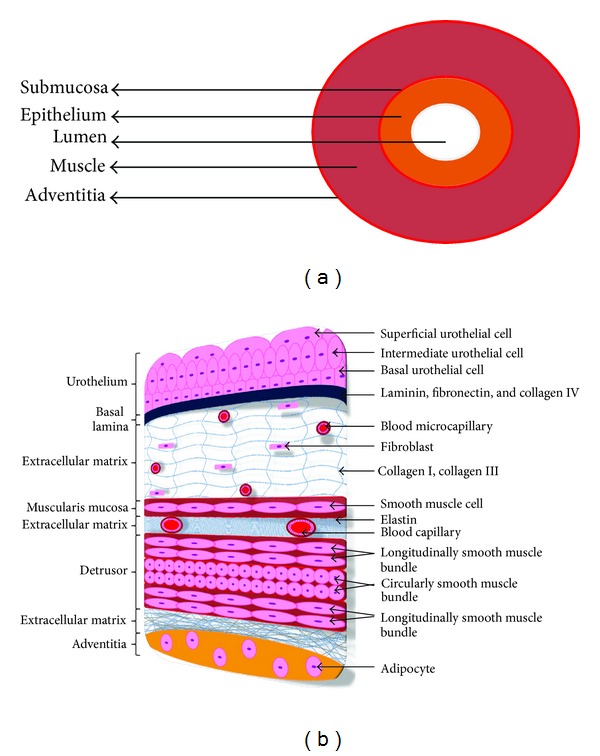
(a) Diagram for general architecture and cell layers of urinary bladder and urethra. (b) Diagram for the histology of the urinary bladder.

Urinary bladder and urethra are consisting of epithelium on the lumen surrounded by a collagen rich connective tissue and muscle layer. The epithelial layer serves as a barrier that prevents the urine from sweeping into the body cavity. The collagen rich layer and muscle tissue surrounding the epithelium maintain the structural integrity of the organ and contract to transport or expel the urine (Figure 1(a)).

*Bladder Anatomy.* Briefly, the bladder consists in four distinct layers (Figure 1(b)): the adventitia, the muscular layer, the submucosa layer, and, finally, the urothelium [13]. The muscle layer is called detrusor muscle and its contraction allows the expulsion of urine to the outside. The submucosa is a connective tissue joining the detrusor and the urothelium and it is important to maintain a well-organized and functional epithelium. It is mainly constituted of collagen types I and III fibres, elastic fibres, and unmyelinated nervous endings [14, 15]. The bladder epithelium is transitional; all the urothelial cells are attached on the basal lamina composed of ECM (collagen IV and laminin). Urothelium consists of the basal cells, intermediate cells, and umbrella cells. The basal cells are the progenitors and very low differentiated cells. The umbrella cells are the most superficial and differentiated type of urothelial cells. Umbrella cells organize at their surface a protein complex specific to the urothelium, the uroplakin plaque, which is the terminal marker of urothelial differentiation. Uroplakins and tight junctions between cells assure the impermeability of the bladder [16, 17].

*Urethral Anatomy. *The urethra is a tubular structure composed of multiple layers of tissues [13]. The structure of the tube is roughly similar to the one of the bladder: smooth muscles with intrafascicular connective tissue, submucosa, or lamina propria with collagen fibres and microvascularization, and, finally, the urethral epithelium lying on the basal lamina. The epithelial lining of the anterior urethra of males consists of stratified columnar epithelium except at fossa navicularis where it becomes stratified squamous epithelium. The epithelial lining of the female urethra is transitional in the proximal one-third and nonkeratinized stratified squamous in the distal two-thirds [1].

## 3. Armamentarium for Tissue Engineering of Bladder and Urethra

Huge similarities in the basics of tissue engineering between these organs exist. Because of these similarities in the choice of the scaffold and seeded cells between bladder and urethral reconstruction, the experience gained from bladder engineering can be harnessed to guide the urethral regeneration and vice versa.

### 3.1. Cell Sources

Tissue specific autologous cells harvested from an individual, cultured ex vivo to be expanded, and reintroduced into a second site for repairing damaged tissue with “self” are ideal for tissue engineering of bladder and urethra.

#### 3.1.1. Progenitor Cells

These cells reside within each organ, have limited self-renewal capacity, and differentiate into only one defined cell type. They are responsible for new cell differentiation and tissue formation during the normal process of tissue regeneration due to natural turnover, aging, and tissue injury [18].

*Epithelial Cells*. (1) Autologous urothelial cells (UCs): classically, these cells are obtained from urinary bladder and have often been used in urethral and bladder reconstruction. Many successful protocols have been developed for urothelial cell cultures from small bladder biopsies [19]. However, this may involve surgical intervention and trauma to urogenital tract. For these reasons, researchers have developed urothelial cell isolation techniques that are less invasive to obtain autologous urothelial cells from urine [20] and bladder washes [21].

(2) Autologous epidermal cells: these cells can be harvested from penile foreskin because of its abundant resources. When these cells were seeded on acellular collagen matrix and implanted in rabbits, urethrography showed wide urethral caliber maintained without any sign of strictures and histology revealed transitional cell layer [22]. However, this approach may have the problems of nonavailability in case of circumcision and surgery and nonspecificity of the used cells.

(3) Autologous oral keratinocytes, such as buccal keratinocytes and lingual keratinocytes, have also been used as a source of epithelial cells [23]. However, a biopsy from buccal mucosa to harvest epithelial cells in the presence of less invasive methods would add morbidity to the procedure.

*Smooth Muscle Cells (SMCs)*. Autologous SMCs offer the potential for improved ECM compliance and tissue elasticity, in addition to angiogenesis and epithelial maturation. In bladder, SMCs are essential to allow for contraction of the engineered tissue for urine expulsion [24, 25].

#### 3.1.2. Stem Cells

Stem cells are undifferentiated cells that have self-renewal potential and are able to differentiate into mature nonregenerative cells and effector cells [26]. Stem cells can be the source of cellular component for engineered UB or urethra when progenitor cells from diseased or malignant UB may not be appropriate for engineered constructs [27, 28]. Although embryonic stem cells can be a source of urothelial cells, their use is limited due to ethical considerations, malignant potential, and problems with cell regulation. Adult stem cells derived from bone marrow (BM), adipose tissue, and urine can avoid these problems [29]. Bone marrow stem cells (BMSCs) can be differentiated into SMCs and UCs [30, 31]. Nevertheless, their use is restricted because of low content of stem cells in BM, prolonged time of in vitro expansion, and patient discomfort during BM aspiration. Adipose-derived stem cells (ADSCs) are present in abundant quantities, harvested by a minimally invasive procedure, and differentiated along multiple cell lineage pathways in a regulated and reproducible manner [32]. ADSCs have been successfully differentiated into UCs either with all-trans-retinoic acid or coculture with UCs [33, 34]. Human ADSCs were differentiated into SMCs and seeded on composite UB scaffold. These seeded scaffolds maintained bladder capacity and compliance when implanted in nude mice [35]. A subpopulation of urine-derived stem cells (USCs) has been identified with the capacity for multipotent differentiation. These cells expressed pericyte and mesenchymal stem cell markers [36]. Upon induction with appropriate media in vitro, USCs were differentiated into bladder-associated cell types, including functional urothelial and smooth muscle cell lineages [37].

### 3.2. Scaffolds

Scaffolds facilitate the delivery of cells to desired sites in the body, define a three-dimensional space for the formation of new tissues with appropriate structure, and provide mechanical support for the newly regenerated tissues [38]. The selected biomaterial should be biodegradable and bioresorbable to support the reconstruction of a completely normal tissue without inflammation. Such behavior of the biomaterials avoids the risk of inflammatory or foreign-body responses that may be associated with the permanent presence of a foreign material in the body. The degradation products should not provoke inflammation or toxicity and must be removed from the body via metabolic pathways. The degradation rate and the concentration of degradation products in the tissues surrounding the implant must be at a tolerable level [39].

Ideal biomaterial for bladder and urethral regeneration should allow for even and constant attachment of mature epithelial cell layer on the luminal surface and harbor multiple cell layers of smooth muscle cells on the outside. It should also provide adequate mechanical support and prevent collapse prematurely before new tissue formation in vivo [40].

Two main types of biomaterials or scaffolds exist: synthetic and natural.

#### 3.2.1. Synthetic Scaffolds

Synthetic polymers such as the biodegradable polymers poly(glycolic acid) (PGA) and poly(lactic acid-co-glycolic acid) (PLGA) are biomaterials made of macromolecules assembled with covalent links. The main advantage of synthetic polymers is the capacity to manufacture any forms of organ in three dimensions, in a quantitative and reproducible way, and at relatively low cost. Because it is an artificial material, it eliminates the problems related to tissue harvesting. Moreover many characteristics can be controlled such as porosity and mechanical properties. These biomaterials are degraded by hydrolysis and fragments removed through metabolic pathways [41–43]. These polymers contain none of the molecular signals that are so relevant in directing cell activity and fate.

#### 3.2.2. Biologically Derived Scaffolds

They are chemically and mechanically decellularized tissues (such as small intestinal submucosa (SIS), bladder acellular matrix (BAM)). They have the advantage of providing inherent bioactivity and mechanical similarity to native ECM due to the inherited presence of growth factors and ECM proteins [44]. However, a major disadvantage of these systems is the routine variability in protein composition among batches. There may also be ethical issues regarding their availability, although most naturally derived scaffolds are porcine xenografts with the possibility of disease transmission.

*Alternate Strategy: Self-Assembled Engineered Tissue*. Even if the acellular matrices they are submitted to a decellularization and a sterilization process, exogenous ECM materials still retain a significant portion of residual DNA that could affect biocompatibility [45]. The self-assembly method is able to produce a tissue built by the cells themselves where a dense ECM is completely produced by fibroblasts. In opposition to all exogenous scaffolds models, these models are autologous, which is a real advantage by eliminating the biocompatibility concerns. The absence of immunological response should reduce the inflammatory and fibrotic reactions and consequently improve the success rate of the procedure. For several years now, this method has been explored for the reconstruction of urologic tissues [46–52]. The self-assembly technique is a tissue engineering method developed for burn patients. Nevertheless, it proves to be useful for tissue reconstructions ranging from skin to blood vessels [53–55]. In the self-assembly method, cells receive right signalling for their appropriate differentiation. Then, it results that the transplanted engineered tissue is very similar to the one that has to be replaced.

Cells must be extracted from the patient biopsy. In order to minimize the invasiveness character of this step, several techniques were developed to maximize quantity and purity of cells, which could be obtained from a biopsy [51, 53, 56]. Fibroblasts are extracted from dermis or oral mucosa, adipose-derived stromal cells from hypodermis, and smooth muscle cells and vesical fibroblast cells from bladder.

Classically, dermal fibroblasts (DFs) were isolated from a human skin. They were seeded in a tissue culture plate containing a custom-built tissue-anchoring device. DFs were cultured in DMEM supplemented with 10% FBS and antibiotics. Sodium L-ascorbate was added to the culture medium to stimulate ECM synthesis. DFs were cultured 21 days until they proliferated over the tissue-anchoring device and their neosynthesized ECM proteins had self-assembled into an adherent living tissue sheet. The self-assembled matrix is formed with a highly organized matrix of collagen types I and III; both are the most common types of collagen in the bladder. Moreover, when seeded with UCs, the presence of laminin suggested the formation of a basal membrane under UCs [46].

Ideal biomaterial for bladder and urethral regeneration should allow for even and constant attachment of mature epithelial cell layer on the luminal surface and harbor multiple cell layers of smooth muscle cells on the outside. It should also provide adequate mechanical support and prevent collapse prematurely before new tissue formation in vivo. The self-assembled engineered sheet possesses many of these favorable characteristics. Urothelial cells when seeded dynamically on self-assembly collagen sheets formed well-stratified urothelial cell layer that exhibited uroplakins as indicator of terminal differentiation of the cells. When permeability test was performed using Franz-type diffusion cells, no significant difference between cumulative 14C-urea permeation of this tissue-engineered graft and native tissue was observed [46]. When mechanical properties of the engineered tissues were analyzed by uniaxial tensile testing, the engineered self-assembled tissues exhibited mechanical characteristics similar to native tissues [51]. These engineered tubes showed mean burst pressure higher than normal porcine urethra especially after pulsatile stimulation in bioreactor for 1 week. These tissues did not tear where the stitches were made. After joining together two full sections of these grafts, 14 cm long tubular graft was obtained. When submitted to pressure, the freshly sutured constructs leaked at high internal pressure of approximately 40 mmHg (54 cm H_2_O) [51]. A self-assembly approach provided excellent results regarding biological functions and cell differentiation because it closely respected the microenvironment of cells. A major concern is that this technique was time consuming for producing tissue equivalents with enough ECM to allow manipulations. Since the time needed to produce engineered tissue is critical, the matrix deposition rate was enhanced without inducing fibroblast hyperproliferation and tissue fibrosis. Addition of lysophosphatidic acid, a natural bioactive lipid, increased the rate of collagen deposition, but it did not modify the amount of secreted collagen [57].

Self-assembled collagen sheets were also made from human adipose-derived stromal cells with similar mechanical and architectural characteristics similar to those driven from human fibroblasts. However, they did not sustain the development of a differentiated and functional urothelium when urothelial cells were seeded which necessitated addition of fibroblasts to help the formation of water-seal epithelial layer [58].

## 4. Tissue Engineering of Urethra

The ideal engineered urethral substitute should be taken well after implantation, do not undergo contraction, fibrosis, or rejection. It should be also be impermeable, cheap and have good handling characteristics [59].

**Figure 2:**
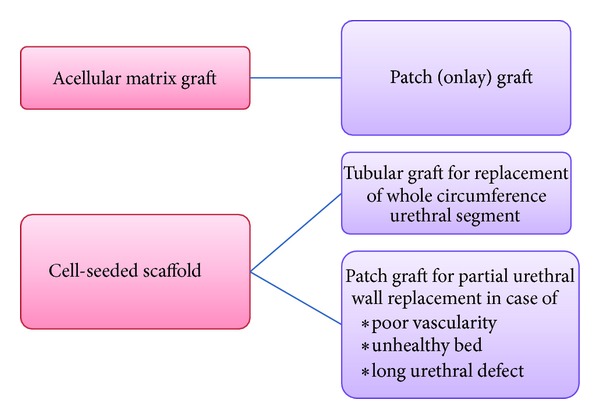
Strategies for urethral replacement using tissue engineering techniques.

*Strategies for Urethral Tissue Engineering*. Tissue engineering approaches include the use of acellular matrices and cell-seeded matrices (Figure 2). Many acellular matrices have been used in many animal and clinical trials to replace urethral segments. They function as scaffolds to guide urothelial and connective tissue regeneration. Acellular graft can be used only when a healthy part of urethral wall exists; tissue regeneration sweeps from its edges to complete urethral lumen. In animal studies, SIS grafts encouraged regeneration of the normal rabbit epithelium supported by a collagen and smooth muscle tissue when used as only urethral graft [60].

A number of human studies have used cadaveric bladder submucosa: 2 studies in patients with urethral stricture and one in hypospadias [61–63]. In a comparative study of 30 patients with urethral stricture, bladder submucosa graft was as successful as buccal mucosa in patients with a healthy urethral bed, no spongiofibrosis, and good urethral mucosa [62]. In the hypospadias study, collagen matrix has been used as onlay graft for the repair of hypospadias in four boys. The created neourethras ranged from 5 to 15 cm long with a successful outcome in regard to cosmetic appearance and function [63]. SIS is a readily available acellular matrix with long-term safety and efficacy. It has been used in substitution urethroplasty for urethral stricture in many human studies [64–66]. In a study with a long-term follow-up period (71 months), SIS urethroplasty was successful in 19 patients (76%) and 6 (24%) were failures (100% when the stricture length was more than 4 cm). The clinical outcome was considered a failure when any postoperative instrumentation, including dilation, was needed [66]. Therefore, in general, acellular matrices can be used only as an alternative option in patients with short-to-medium urethral defect with healthy urethral bed and no or minimal spongiofibrosis.

Cell-seeded constructs have been adopted as the strategy of choice for replacement of tubularized urethra segment. They have been used successfully both experimentally in large animal models and clinical trials. Two studies on rabbits used cell-seeded constructs to replace full thickness urethral segments. Both studies used bladder submucosa matrix, seeded with UCs in one study and foreskin epidermal cells in the other. Urethral lumen was patent with the seeded grafts in comparison to matrix alone [22, 67]. In a preclinical study in large animal model with long urethral segment substituted, 6 cm of anterior urethra was excised and replaced with porcine bladder submucosa seeded with autologous UCs and SMCs from UB. After one year of followup, computed tomography (CT) urethrography showed patent urethra with wide caliber and gross examination revealed healthy urethral mucosa with no diverticulum formation. Histological examination showed that the implanted cells survived after surgery and contributed significantly to the well-formed urethral wall [68]. Three human studies with cell-seeded constructs were carried out, one in adult patients with lichen sclerosis [23] and 2 in children for complex posterior urethral defects and hypospadias [69, 70]. In the adult study, autologous keratinocytes and fibroblasts were isolated from buccal mucosa biopsy and seeded on donor acellular dermis. This graft was implanted either as a one- or a two-stage procedure. One patient needed complete excision while another patient required partial excision. Three patients have patent urethra after some form of urethral instrumentation. This high failure may be attributed to the underlying pathology and graft contracture [23]. In the posterior urethroplasty study, a tissue biopsy from each of the 5 children was taken and epithelial and muscle cells from each patient were isolated, then seeded on tubularised polyglycolic acid:poly(lactide-co-glycolide acid) scaffolds to be implanted in posterior urethra. Tubularized seeded urethras remained patent in all patients for 6 years [69]. In the hypospadias study, autologous urothelial cells from bladder washes of 6 children were isolated and seeded on acellular dermis to form cell-seeded constructs. These constructs were used to repair severe hypospadias defects in 6 children. Ultimately, five patients void in a standing position while the last patient developed stricture that was managed successfully with internal urethrotomy [70]. However, this cell-seeded constructs technique would be preserved for complicated cases like cripple hypospadias, bladder exstrophy, and complex and long urethral stricture.

**Figure 3:**
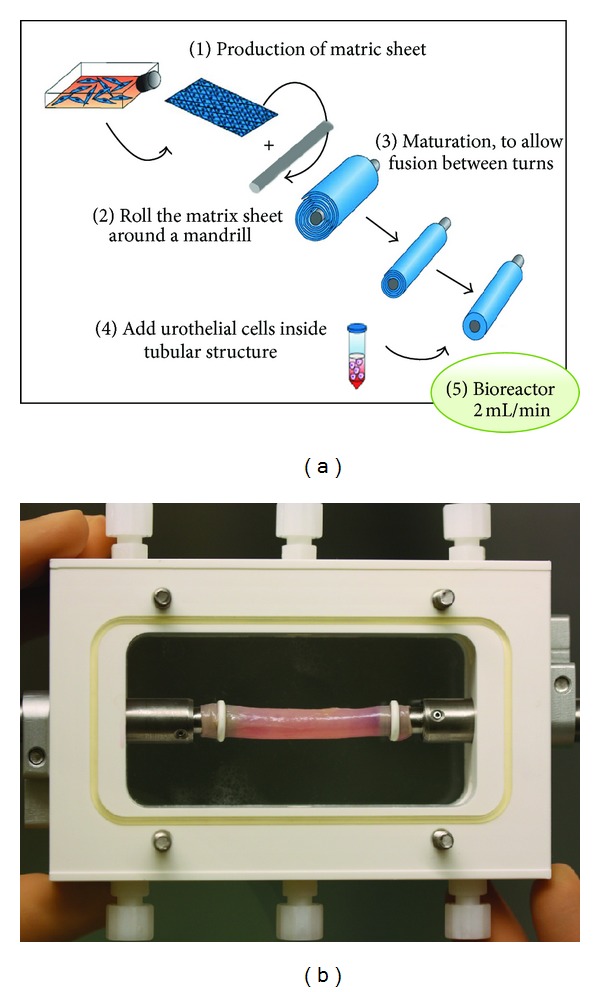
Production of cell-seeded tubular urethral graft using self-assembly technique. (a) After the production of a matrix sheet by fibroblasts, it is rolled to form a tube and urothelial cells are seeded in the lumen. (b) Bioreactor for tubular cell-seeded grafts to stimulate differentiation and formation of watertight mucosal layer.

**Figure 4:**
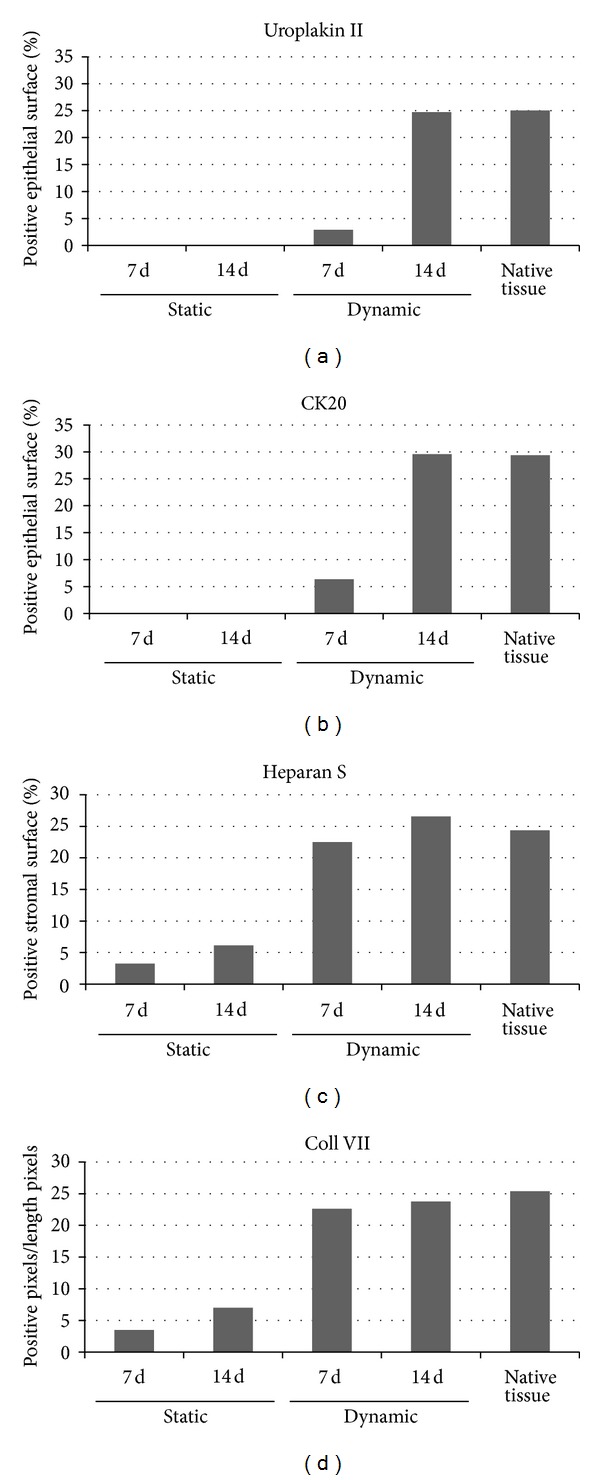
Mechanical stimuli-induced urothelial differentiation in a human tissue-engineered tubular genitourinary graft. Data from immunofluorescence were raised against the indicated molecule as presented in Cattan et al. [48]; they were analyzed with NIH ImageJ software. In (a) percentage of uroplakin II positive surface relative to the urothelial surface is depicted without stimulation (static) at day 7 (7 d), or day 14 (14 d), or with mechanical stimulation (dynamic) and compared with a native porcine tissue. In (b) similar data are presented for cytokeratin 20 (CK20). In (c) heparan sulphate (Heparan S) positive surface was evaluated compared to stromal surface. In (d) data depict collagen VII (Coll VII) positive pixels relative to the length in pixel of the basal lamina.

Decellularized nonautologous matrices with nonstratified urothelium have been implanted in the previous studies. A new construct with scaffold and well-stratified cell layers formed by the patient own cells would be a great advantage and optimization towards safe and more efficient urethral replacement. Using self-assembly method, collagen sheets from DF were produced in the presence of ascorbic acid and then rolled in a tubular structure (Figure 3). Mechanical characteristics of this model are roughly similar or even better than the ones of the native tissue [51]. Tubes were placed in constant flow to stimulate the differentiation of the seeded urothelial cells [48]. Evidence of terminal differentiation of urothelial cells including uroplakins was expressed and formed near native structures (Figure 4). These engineered tubular constructs withstand suturing and do not break. When these constructs were implanted subcutaneously in vivo in nude mice, it was found that urothelial cells survived and mature multilayer epithelium with well-formed subepithelial collagen layer was produced. When endothelial cells were incorporated into such constructs, it was found that these endothelialized grafts have earlier vascularization than nonendothelialized grafts, which could decrease the necrosis of the transplanted tissue in early period after in vivo implantation [71].

## 5. Tissue Engineering of Urinary Bladder

Since UB is a temporary urine reservoir, water tightness and compliance are two main required features in the future substituted tissue. Bladder must accommodate various volumes of liquid without a significant increase in pressure, which could be damageable to the kidneys [72]. This role is mainly due to the presence of muscle layer and elastic fibres [73]. Bladder must also be a safe storage for the toxic components of urine preventing their reabsorption into the circulation during storage. This is the role of bladder sphincter and watertight epithelium [16]. The engineered bladder should also contract to expel the urine outside preventing its retention inside. The generation of a complete bladder wall requires not only a multilayer cellular scaffold but also vascularization and innervation of the united smooth muscle structure to be regenerated. The addition of growth factors (nerve growth factor in combination with vascular endothelial growth factor, e.g.) might enhance the regeneration of acellular matrix [74].

**Figure 5:**
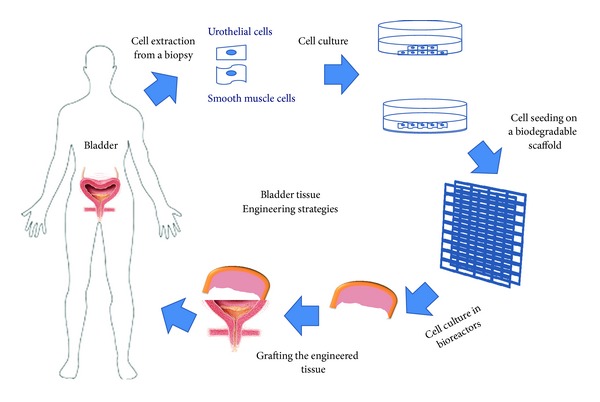
General strategy for tissue-engineered urinary bladder.

*Strategy for Bladder Tissue Engineering*. When gastrointestinal tissue is in contact with the urinary tract, multiple complications may ensue, such as increased mucus production, infection, urolithiasis, perforation, metabolic disturbances, and malignancy. Therefore, numerous investigators have searched for novel techniques for bladder replacement using cell-seeded scaffolds (Figure 5).

A nonseeded scaffold technology can be theoretically the ideal strategy for bladder replacement as it is simple and does not require cell harvest and in vitro culture. These scaffolds, when implanted, were thought to enhance tissue regeneration and recruit the local and systemic stem cells to the site of implantation to contribute in the new tissue formation. For this to occur, these scaffolds should imitate the natural ECM to orchestrate the different steps involved in the regeneration process. That is why naturally derived ECM matrices were the first to be used for this approach. SIS and BAMG were widely explored in experimental studies [75–77]. However, nonseeded scaffolds failed to show full regeneration of the bladder wall. According to some studies, only approximately 30% of the smooth muscle layer was able to grow back [78]. The failure of cell-free scaffolds to replace bladder can be attributed to many factors. These include extensive scarring within the graft due to xenographic or nonautologous nature of the graft and early exposure of the scaffold to urine, which induce scarring. Urine also was toxic to the recruited progenitor and stem cells. Additionally, the lack of muscle cell layer decreases the elasticity of the wall and prevents the bladder contraction and cycling.

In large animal model [79], a subtotal cystectomy was followed with subsequent replacement with a tissue-engineered organ. Three groups were included in this study; subtotal cystectomy only, subtotal cystectomy with subsequent replacement with nonseeded scaffolds, and subtotal cystectomy with subsequent replacement with a tissue-engineered graft. An average bladder capacity of 95% of the original precystectomy volume was achieved in the tissue-engineered bladder replacements while cystectomy-only and nonseeded controls maintained average capacities of 22% and 46% of preoperative values, respectively. These findings were confirmed radiographically. The compliance of the tissue-engineered bladders showed almost no difference from preoperative values that were measured when the native bladder was present (106%). Subtotal cystectomy reservoirs that were not reconstructed and the polymer-only reconstructed bladders showed a marked decrease in bladder compliance (10% and 42% of total compliance, resp.). On histological examination, the nonseeded scaffold bladders presented a pattern of normal urothelial cells with a thickened fibrotic submucosa and a thin layer of muscle fibers. The retrieved tissue-engineered bladders showed a normal cellular organization, consisting of a trilayer urothelium, submucosa, and muscle. Preliminary clinical trials for the application of this technology have been performed [80]. In these preliminary studies, seven patients with neurogenic bladder due to myelomeningocele were identified as candidates for cystoplasty. A bladder biopsy was obtained from each patient. Urothelial and muscle cells were grown in culture and seeded on a biodegradable bladder-shaped scaffold made of collagen or a composite of collagen and polyglycolic acid. Three groups were included; cell-seeded collagen matrix engineered bladders without the omental wrap in 3 patients, a cell-seeded collagen matrix bladder with a full omental wrap in 1 patient, and cell-seeded composite collagen-PGA bladders wrapped with omentum in 3 patients. Postoperatively, the mean decrease in bladder leak point pressure at capacity and the mean increase in volume and compliance were greatest in the composite engineered bladders with an omental wrap (56%, 1.58-fold, and 2.79-fold, resp.). No metabolic consequences were noted, urinary calculi did not form, and renal function was preserved. The engineered bladder biopsies showed an adequate structural architecture and phenotype.

Two multicenter phase II clinical studies, the first involving pediatric patients with neurogenic bladder secondary to spina bifida and the second involving adult patients with neurogenic bladder secondary to spinal cord injury, were then undertaken to evaluate the safety and effectiveness of the tissue-engineered Neo-Bladder Augment (NBA). The pediatric study included 10 patients at 4 medical centers in the United States, requiring augmentation cystoplasty due to bladder pressures ≥40 cm H_2_O and/or development of upper urinary tract changes. After an open bladder biopsy, autologous cells derived from the biopsy were expanded in vitro and seeded onto the NBA scaffold. This construct was then implanted into each patient. The procedure was well-tolerated and 6 of the 10 patients showed clinical improvement based on urodynamic studies, radiography, and voiding diary results. The adult study involved 6 patients with severe bladder dysfunction secondary to spinal cord injury. The same procedure was performed to create the implantable construct from the patients' cells and the NBA scaffold, and after 2 years of followup, it was found that 4 of the 6 patients responded well to the tissue-engineered bladder. In both studies, the implants of patients who were able to undergo normal bladder cycling (filling and emptying) regenerated well, while those implanted in patients who did not have normal bladder cycles due open bladder necks or other physiological issues did not respond as well to this therapy. This further supports the idea that conditioning engineered bladder tissue within a specially designed bioreactor prior to implantation may lead to improved clinical results [81].

**Figure 6:**
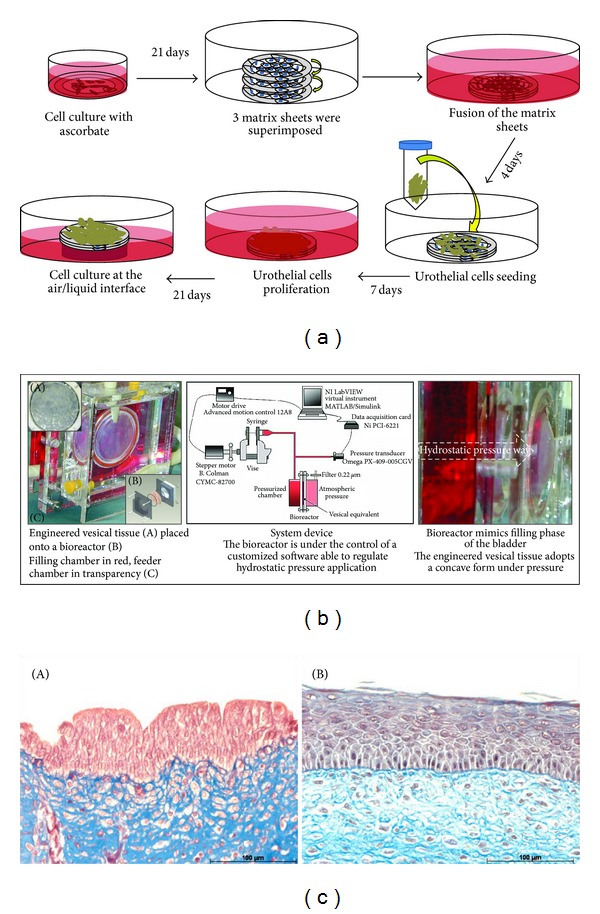
Production of a vesical equivalent by the self-assembly technique. Vesical equivalent reconstruction can be divided in two major steps. (a) Upper panel: deposition of extracellular matrix to create a manipulatable sheet followed by seeding of urothelial cells. (b) Middle panel: proliferation and differentiation of urothelial cells using a specially designed bioreactor. Bioreactor mimics filling and emptying phases of the bladder. Note that under pressure, vesical equivalent adopts a concave form. (c) Lower panel: self-assembly technique allows production of human reconstructed endothelialized vesical equivalent. (A) Masson's trichrome staining of a slice from a native porcine bladder. (B) Masson's trichrome staining of a slice from a human reconstructed vesical equivalent.

*Formation of Vesical Equivalent with Self-Assembly Technique*. After cell extraction, the bladder substitute can be produced (Figure 6). Stromal cells are cultivated in the presence of ascorbate to enhance collagen synthesis, secretion, and deposition in order to constitute the extracellular matrix. After three weeks, urothelial cells are seeded onto the top of the stromal component, preassembled three sheets of fibroblasts. Cells are allowed to proliferate for one week; then the equivalent is to be cultured at the air/liquid interface for three more weeks. By preconditioning the reconstructed tissue in a dynamic environment to simulate the filling and emptying cycles of the bladder, the use of bioreactor may enhance the mechanical behavior of the extracellular matrix [47]. Moreover, it has been demonstrated that a dynamic environment can lead to a better differentiation of the urothelial cell compared to static one [48]. The flow and pressure seem to stimulate the expression of not only uroplakin II but also CK20, two well-known terminal markers of the differentiation of the urothelial cells [82].

The feasibility of the reconstruction of an autologous human urologic tissue has been demonstrated but, in order to continue to ameliorate the self-assembly technique adapted to the bladder reconstruction, the use of adipose-derived stromal cells could be investigated. They are fairly easy to obtain and a small biopsy could generate a large number of adipose-derived stromal cells [83]. These cells have been described to be able to secrete mediators, which are essential to the endothelial cell culture and then vascularization of the graft, such as vascular endothelial growth factor (VEGF), hepatocyte growth factor (HGF), fibroblast growth factor (FGF), and the stromal cell derived factor-1 (SDF-1) [84]. SDF-1 is a mediator that could be linked to the formation of new capillaries by attracting endothelial cells [85]. The angiogenesis is a key factor for the graft takes. This complex process allows the supply of the graft with nutrients and oxygen, then reducing necrosis, fibrosis, and apoptosis of transplanted cells [86, 87]. Additionally, these cells have shown the potential to decrease immune response of Th2 in a respiratory model. Even after an activation of the immune response by TNF-*α*, the conditioning medium of the adipose-derived stromal cells had an anti-inflammatory effect over U937 cells [87, 88]. A subpopulation of stem cell exists in the adipose-derived stromal cell populations and is evaluated around 2% of the total cells. These cells add plasticity to the self-assembly model and, in a very interesting manner, adipose-derived stem cells could differentiate mostly in smooth muscle cells after transplantation. All those advantages need to be investigated further and they could be crucial for the graft take and function of a reconstructed tissue.

## 6. Existing Challenges and Future Directions

The main issues preventing the development of larger bladder contractile tissues that allow physiologic voiding include the development of correct muscle alignment, proper innervation, and vascularization. Patients who need bladder replacement have either diseased or malignant bladders and their bladder cells may be abnormal; hence we need a suitable source of cells that may be easily harvested and differentiated to become normal urinary bladder cells. These cells will need to be primed and driven to regenerate the main bladder cell constituents (smooth muscle and urothelium) [89]. For these purposes, cells from upper urinary tract may be a good alternative [90]. Amniotic fluid stem cells first introduced by Atala have emerged as a potential source of stem cells [91]. They have been recently differentiated into urothelial cells and SMCs [92, 93]. Another potential exists for demucosalisation of bowel coupled with urothelial cell seeding. A new extraluminal technique for demucosalisation was developed to remove deep colonic epithelial crypts to prevent the possibility of regrowth of colonic epithelium and at the same time maintain sterility by eliminating the opportunity of contamination [94].

Bioreactors should be thoroughly investigated to explore their role in final SMCs maturation, alignment, and functional contraction.

## 7. Conclusions 

Tissue engineering is an evolving field of research for organ and tissue replacement and disease investigation for many pathologies affecting the human being. Cell-seeded scaffolds are considered the optimized strategy for engineering autologous substitutes for bladder and urethra. At the moment, self-assembly method, because of its unique exogenous material-free feature, seems to be the closest to physiological conditions among the available scaffolds, raising the possibility to generate more appropriate tissue for surgical reconstruction. Bioreactors could improve cell viability, proliferation, and maturation by preconditioning the cell-seeded scaffolds in a simulated physiologic environment. This eventually helps the formation of well-differentiated and functional tissue substitutes. Although there is a considerable improvement in tissue engineering of these 2 hallow organs, there are some hurdles for the fully optimized graft including stimulating the implanted muscles to contract, vascularity, and nerve supply.
